# Role of Immunotherapy in Conjunction With the Surgical Treatment of Breast Cancer: Preoperative and Postoperative Applications

**DOI:** 10.7759/cureus.71441

**Published:** 2024-10-14

**Authors:** Nagma Sabu, Hussein Attia Hussein Mahmoud, Juan Felipe Salazar González, Nithin Naruboina, Samuel Esteban Rojas Prieto, Seyanne Govender, Vegunta Ruthvik Phani Narayan, Bhalala Priyank Batukbhai, Yasmin Ahmadi

**Affiliations:** 1 Department of Surgery, University of Perpetual Help System Dalta - JONELTA Foundation School of Medicine, Las Pinas, PHL; 2 Diagnostic Radiology, Heliopolis Hospital, Cairo, EGY; 3 General Medicine, Clínica Renovar, Villavicencio, COL; 4 General Practice, Banaras Hindu University, Varanasi, IND; 5 General Practice, Universidad Nacional de Colombia, Bogotá, COL; 6 General Practice, American University of the Caribbean, Cupecoy, SXM; 7 General Practice, NRI Medical College & General Hospital, Mangalagiri, IND; 8 Internal Medicine, Grodno State Medical University, Grodno, BLR; 9 School of Medicine, Royal College of Surgeons in Ireland - Medical University of Bahrain, Muharraq, BHR

**Keywords:** cancer immunotherapy, immunomodulators, immunotherapy-related adverse events, metaplastic breast cancer, preoperative immunotherapy for breast cancer

## Abstract

Breast cancer is one of the most common cancers in the world. Since the appearance of molecular medicine, the perspective of breast cancer treatment has changed, making it more successful in comparison with the treatment during previous years. Numerous ongoing trials are exploring the capacity of immunotherapy, mainly in immune checkpoint inhibitors (ICIs), in conjunction with conventional therapies or with antibody-drug conjugates (ADCs). The current narrative review discusses the advantages and limitations of immunotherapy in breast cancer treatment in conjunction with the surgical options available. Going through the modern capacity of surgery treatment and how the use of immunotherapy in conjunction with it has emerged as a transformative approach to breast cancer and listing the main complications and adverse effects caused by ICIs. We searched Google Scholar, PubMed, MEDLINE, and EMBASS. Fourteen different articles showed that the use of cytokines and cancer vaccines revealed new possibilities to treat breast cancer with antibodies against PD-1/PD-L1 (pembrolizumab), PI3K/Akt/mTOR (alpelisib and everolimus), CAR T-cell (chimeric antigen receptor), PARP (poly ADP-ribose polymerase), and CTLA4 (cytotoxic T-lymphocyte-associated protein 4), and with representative relevance of changing in tumor microenvironment. Immunotherapy made it possible to reduce recurrences, after radiotherapy and surgery. Estrogen receptor (ER) and human epidermal growth factor receptor 2 (HER2) targets show also a high effectivity. In recent years, the release of new strategies has become promising, for changing the microenvironment and de-escalation of therapy based on tumor biology, novel biomarkers, and tumor spread.

## Introduction and background

Breast cancer is one of the most prevalent forms of cancer, affecting millions of people worldwide [1]. Immunotherapy treatment strategies provide the potential to improve the delivery of anti-cancer agents into breast cancer cells, with the possibility of improving patient outcomes [2]. Previously, breast cancer treatment has relied on surgery, chemotherapy, and radiation therapy. However, due to the limitations of these methods, such as poor target selectivity, drug resistance, and challenges in managing metastatic disease, an increasing need for exploring the potential of immunotherapy has been felt [3-6].

Immunotherapy therapies targeting proteins such as HER2, pathways such as PI3K/AKT, and inhibitors targeting these pathways have been instrumental in the progress of breast cancer treatment [7]. Using novel treatment strategies in conjunction with immunotherapy, which can be engineered to target characteristics specific to tumors, such as their distinctive microenvironment, and deliver therapeutic agents more effectively, could improve breast cancer treatment [8,9].

Immunomonotherapy has especially progressed treatment for triple-negative breast cancer (TNBC) and PD-L1-positive cases [10,11]. In addition, antibody-drug conjugates (ADCs) combined with immune checkpoint inhibitors (ICIs) are being studied for increased effectiveness [12]. According to Jacob et al. [13], dual immunotherapy, combining CTLA-4 and PD-1 inhibitors, is under exploration, while PARP inhibitors, CAR-T therapies, and microRNA-based treatments are showing promise in ongoing research [13,14].

Immunotherapy, which is designed to use the body's immune system to combat cancer, has emerged as a promising adjunct to conventional cancer treatments [15]. According to Vatner et al. [16], the combination of radiation therapy and immunotherapy holds significant promise, as there is evidence that immunotherapy is most effective when used in combination with standard procedures such as surgery. Radiation therapy can act synergistically with immunotherapy, consequently increasing immune responses, inhibiting immunosuppression, and modifying the phenotype of tumor cells, making the cancer cells more susceptible to immune-mediated killing [16].

## Review

Surgical treatments for breast cancer

In the mid-late nineteenth century (Halstedian era), the predominant surgical option for breast cancer was “radical mastectomy.” During that period, the prevailing belief about breast cancer surgery was "more is better," meaning that more extensive local excision of breast cancer was thought to increase the chances of survival and reduce the risk of local recurrences [17]. However, over time, research has revealed that breast cancer is a set of genetically distinct diseases each with a different prognosis. Thus, necessitating personalized treatment plans based on cancer stage, molecular characteristics, and patient preferences. This shift has resulted in the development of various surgical options while emphasizing the importance of multidisciplinary care in breast cancer management [17,18].

Breast-Conserving Surgery (BCS)

Breast-conserving surgery (BCS), typically followed by radiotherapy to prevent recurrence from the margins, is often favored due to its ability to preserve the psychophysical well-being of the patient when compared to mastectomy, which can result in significant physical deformity and associated psychological impact. The effectiveness of BCS is dependent on the relative size of the tumor when compared to the breast and the ability to obtain clear surgical margins. A meta-analysis was conducted to determine which approach offers better overall survival (OS), and it was found that BCS followed by radiotherapy results in better OS compared to mastectomy [18]. Meanwhile, there is an ongoing debate about whether radiation therapy can be safely skipped in certain patients after BCS [19].

Mastectomy

While mastectomies like simple mastectomy and modified radical mastectomy (MRM) can effectively manage larger or more aggressive tumors, it does not fully address the psychophysical well-being of the patient. Mastectomy types such as skin-sparing mastectomy (SSM) and nipple-sparing mastectomy (NSM) address this concern by preserving the skin and nipple-areola complex, allowing for immediate reconstruction options [17]. The decision to opt for mastectomy is often guided by factors such as tumor size, genetic predispositions (e.g., BRCA mutations), and patient choice. The rise in genetic testing has led to a higher incidence of prophylactic bilateral mastectomy with many BRCA carriers opting for this despite having similar survival rates with BCS and radiotherapy.

Axillary Surgery

Axillary lymph node (ALN) evaluation is necessary to evaluate the spread of cancer. ALN dissection also ensures that no cancerous tissue is left behind after the surgery, but ALN dissection leads to lymphedema in up to 25% of women post-surgery, while the incidence drops to below 10% in those undergoing sentinel lymph node biopsy (SLNB) [20]. The decision to do SLNB over ALND is based on the clinical ALN status and whether the patient has received neoadjuvant chemotherapy (NACT). Some argue that sentinel node surgery is insufficient due to its potential for false-negative results, which stem from the limited sampling of lymph nodes. However, at the St. Gallen International Expert Consensus Conference on the Primary Therapy of Early Breast Cancer, the panel decided that SLNB is adequate if three or greater negative sentinel lymph nodes (SLNs) were identified [21]. There is also an alternative for improving the accuracy of SLNB after NACT, which is gaining traction, i.e., targeted axillary dissection (TAD). TAD includes SLNB and the dissection of previously marked core needle biopsy-proven target lymph nodes. This target lymph node is examined to see if the cancer has responded to the neoadjuvant therapy [20].

Adjuvant Treatments and Their Pros and Cons

While adjuvant and neoadjuvant treatments effectively reduce disease-related mortality, they can also lead to an increased risk of mortality from other causes. Chemotherapy, particularly anthracycline and taxane-based regimens, can decrease disease-related mortality by 10-25%, but they also increase non-disease-related mortality by raising the risk of heart disease and leukemia. Similarly, endocrine therapies like tamoxifen and aromatase inhibitors increase the risk of secondary cancers [22]. Radiotherapy causes an increased risk of ischemic heart disease, particularly when the treatment is on the left breast [23]. In addition to heart disease, radiotherapy for breast cancer can also elevate the risk of lung cancer and esophageal cancer. These therapies also have their adverse effects, including systemic toxicity from chemotherapy, skin damage from radiotherapy, and hormonal side effects from endocrine therapy.

Challenges With Existing Surgical Treatment Options

Despite the effectiveness of BCS and mastectomy, TNBCs have a higher loco-regional recurrence (LRR) rate. The LRR rates are higher for TNBC in comparison with ER-positive and HER2-positive breast cancers [24]. Chemotherapy, when used in conjunction with surgery, reduces the risk of recurrence but carries significant side effects and does not guarantee 100% elimination of the micro-metastatic disease. There is an ongoing debate about the uncertainty in decision-making about the use of certain surgical interventions, especially in cases where there is a pathological complete response (pCR) after neoadjuvant therapy. This highlights the need for better treatment approaches [24]. In low- and middle-income countries where access to surgical treatments has its challenges which include resource limitations, the need for advanced technologies, and specialized personnel affecting outcomes [25].

Integration With Immunotherapy

Immunotherapy has shown promise in reducing recurrence by boosting the immune response to cancer cells. This is because of the ability of immunotherapy to target the tumor microenvironment. This is particularly effective in the case of TNBC. In addition, immunotherapy also has the potential to overcome resistance to traditional therapies [26]. Evidence suggests that combining immunotherapy with surgery may improve outcomes by reducing the incidence of recurrence and due to its potential as a neoadjuvant therapy [27]. It presents promising benefits like antitumor immune response and reduced adverse effects compared to traditional chemotherapy (Table 1) [13,22,27].

**Table 1:** Chemotherapy-induced adverse events (AEs) versus immunotherapy-related adverse effects (irAEs) in patients with breast cancer

System	Immunotherapy	Chemotherapy
General	Flu-like symptoms, fatigue	Nausea and vomiting, fatigue
Skin	Dermatitis (49%)	Hair loss
Gastrointestinal tract	Diarrhea/colitis (20%)	Diarrhea and Colitis have also been linked to Irinotecan, despite it not being the primary choice for treating breast cancer
Endocrine	Endocrinopathies such as hypophysitis (10%), thyroid disorders (18%), and adrenal insufficiency (<5%)	Rarely affects endocrine glands
Respiratory	Pneumonitis (<5%)	Rare but can occur with certain drugs
Cardiovascular	Myocarditis (<5%)	Risk associated with anthracyclines (cardiotoxicity)
Hematology	Rare	Primary affects bone marrow causing anemia, neutropenia, and thrombocytopenia
Immunity	Immune modulation	Immune suppression

Role of immunotherapy in breast cancer treatment

**Figure 1:**
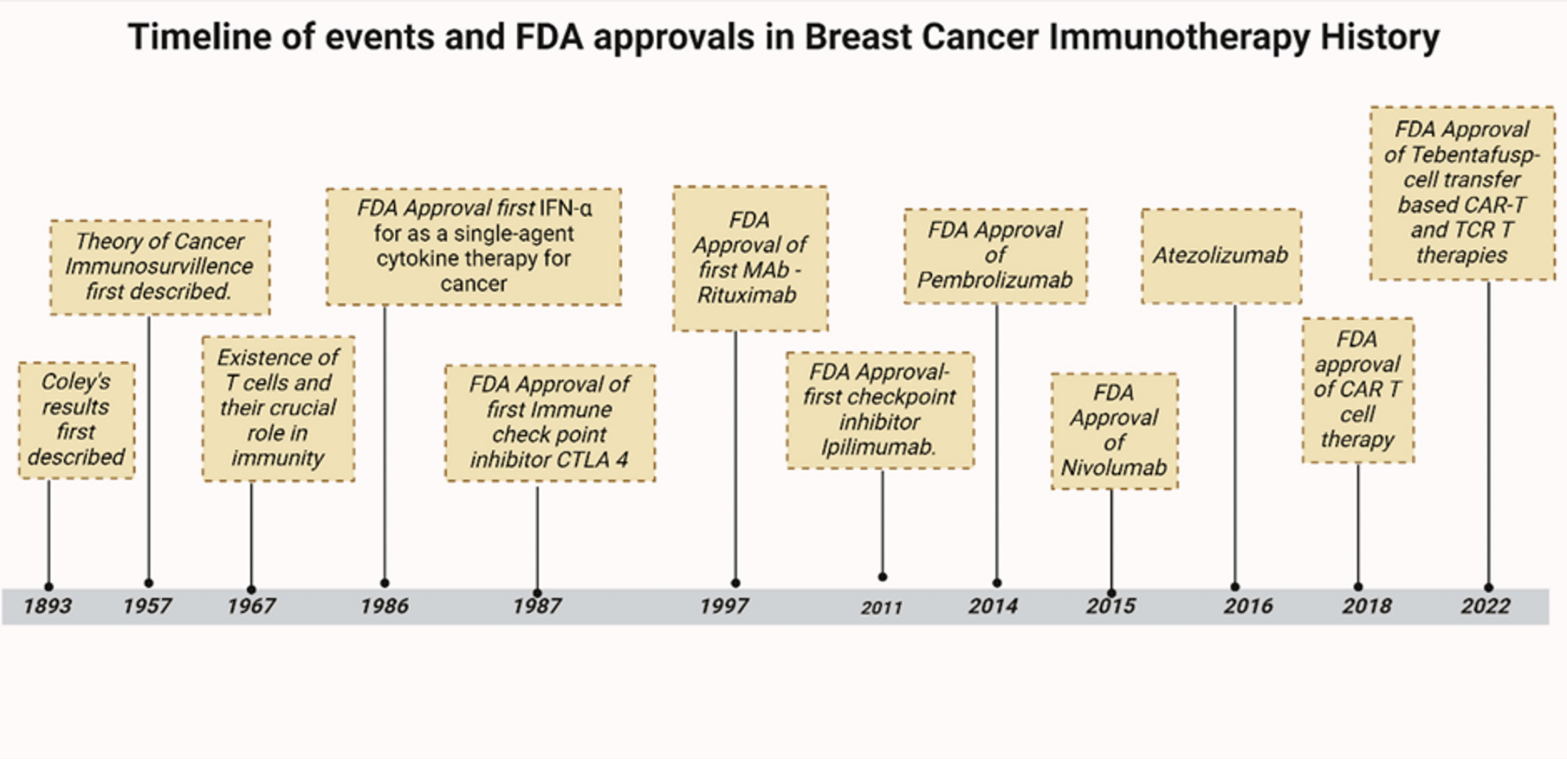
Timeline of events and FDA approvals in breast cancer immunotherapy history Figure made by authors using BioRender.com/t08m311

Immunotherapy has previously been used for cancer treatment. Over the years, many immunotherapeutic strategies have been developed and FDA-approved (Figure 1) [28,29], and currently, there are various immunotherapeutic approaches (Table 2) [28] for breast cancer. The inception of the use of Immunotherapy for the treatment of cancer began when Dr. William Bradley Coley worked on harnessing the immune system to treat bone cancer in 1891 [28]. Even so, it was not until the development of monoclonal antibodies that the role of immunotherapy in the field of oncology gained substantial momentum [28]. In the past, breast cancer was thought to be resistant to ICIs due to its "immune cold" phenotype. However, the discovery of tumor-infiltrating lymphocytes (TILs) and their effect on treatment responses, along with genetic evidence of immune activity in TNBC and HER2-positive disease, has revived attention toward this treatment approach [30].

**Table 2:** Some immunotherapy approaches in breast cancer

Immunotherapy type	Mechanism of action	Challenges
Cytokine therapy	Attempts to enhance immune response through ILs and IFNs.	Relatively low efficacy and high side effects.
PI3K/Akt/mTOR inhibitors	Particularly targets specific signaling pathways to overcome resistance in hormone receptor-positive breast cancer	Tumor microenvironment, complex interactions, and genomic heterogeneity
CAR T-cell therapy	Genetically engineered T-cells to recognize and destroy cancer cells	Found to have limited efficacy in solid tumors, especially the breast
Immune checkpoint inhibitors	Blocks inhibitory pathways that limit T-cell activation	Immune-related adverse events that affect a wide range of body systems
Neoantigen vaccines	Specific and highly personalized vaccines targeting unique tumor-specific antigens	Attempts to overcome immune evasion mechanisms and highly experimental.

The Initial therapies included the use of cytokines like interleukins (ILs) and interferons (IFNs) and cancer vaccines, but these were met with limited popularity due to low efficacy and high side effects [29,31]. The real breakthrough in this field was with the development of ICIs. Monoclonal antibodies targeting PD-1/PD-L1 and CTLA-4 have proven to be valuable tools for overcoming the inhibitory regulation of T-cell activation imposed by tumor cells or the tumor microenvironment (TME) [29]. The newer novel PD 1 inhibitors like pembrolizumab in TNBC seem promising through various research studies. For instance, KEYNOTE-522 trial phase III established that among patients with early TNBC, the percentage with a pathological complete response was significantly higher among those who received pembrolizumab plus NACT than among those who received placebo plus NACT [32].

Chimeric antigen receptor (CAR) T-cell therapy is another promising approach. Although this has revealed tremendous success in hematologic malignancies, its use in solid organ malignancies especially breast cancer has been limited as per the latest studies, and ongoing research aims to overcome the hurdles of antigen heterogeneity and tumor microenvironment (TME) in breast cancer [33]. PI3K/Akt/mTOR inhibitors like alpelisib and everolimus have shown tremendous clinical efficacy, especially in patients with hormone receptor-positive and HER2-negative subtypes of breast cancer by overcoming resistance to traditional therapies already in use and thus improve patient outcomes [34]. However, the PI3K pathways are part of a complex network with numerous parallel cascades. Due to the heterogeneous genomic environment of breast cancer, multiple drivers are often found across different pathways, which means that PI3K-AKT may not always be the primary regulator of mTOR in every cell [34].

Another strategy involves the employment of personalized immunotherapy like neoantigens, unique tumor-specific antigens, and specific vaccines targeting these neoantigens; current research focuses on improving vaccine efficacy, which can help induce a robust and specific immune response to overcome immune evasion mechanisms employed by tumors [35]. In addition, the TME in breast cancer, characterized by its stromal network and dense collagen extracellular matrix, has become a key focus in immunotherapy research. Recent studies have explored how to overcome immunosuppression within this environment to enhance the effectiveness of immunotherapy in breast cancer.

Numerous research efforts have led to the development of new strategies that combine immunotherapy with other treatment modalities. For example, ICIs paired with PARP inhibitors such as olaparib and niraparib have been found to boost antitumor immunity by activating the STING pathway, which promotes T-cell infiltration into tumors. This combination has demonstrated tolerability and improved disease control rates in patients with gBRCA-mutated metastatic breast cancer. Immunotherapeutic strategies like PIK3CG and PD-L1 targeted therapies can be particularly beneficial in the treatment of metaplastic breast cancers, especially in claudin-low breast cancers (CLBCs), which lack traditional treatment targets and can potentially improve patient outcomes [36]. Ongoing clinical trials are aiming at optimizing these combinations further, particularly in TNBC [36]. While there is extensive literature exploring the combination of various therapies with immunotherapy, there is a lack of research exploring Immunotherapy and surgery in conjunction.

Immunotherapy and surgery in conjunction

The two most widely recognized approaches for managing early invasive breast cancer locally are BCS and mastectomy, which may or may not be accompanied by immediate reconstruction. The 10-year rates of local or regional cancer recurrence after BCS, followed by radiation therapy (RT), are about 2-3% for estrogen receptor (ER)-positive and HER-2 positive breast cancers and about 5% for TNBC. These recurrence rates are comparable to those observed after mastectomy in early breast cancer cases. Over time, improvements in systemic treatments have led to reductions in both distant metastasis and local recurrence rates [14].

Chemotherapy could lower the risk of recurrence in early-stage breast cancer by about thirty percent. NACT has the potential to eradicate micro-metastatic lesions, turning inoperable breast cancer into resectable by downstaging the tumor before breast conservation in cases of operable breast cancer. For high-risk patients, including those with large tumor sizes, nodal involvement, low hormone receptor (HR) expression, younger ages, and lympho-vascular invasion, NAC or adjuvant chemotherapy (AC) should be administered [37].

Reducing the chance of cancer recurrence is the goal of chemotherapy administered following radiotherapy or surgery. The goal of targeted medication therapy is to target the proteins in cancerous cells that facilitate their development, metastasis, and proliferation [38]. Treatments for breast cancer that target only the ER and HER2 receptors are the most effective [39].

Because of their roles in multiple carcinogenesis pathways, including the cell cycle, angiogenesis, and metastasis, inhibitors targeting HER2, PI3K, AKT, fibroblast growth factor receptors (FGFRs), mTOR, PARP, or vascular endothelial growth factor (VEGF) may be used as therapeutic methods to halt the advancement of breast cancer [7]. Studies that have already been conducted investigating the use of immunotherapies with surgery have shown that despite the therapeutic benefits, this combination approach has downsides [40]. Cancer patients with solid tumors may benefit from additional protection against recurrent disease if they get perioperative immunotherapy in addition to routine surgery. Many cancer surgery patients may benefit from improved survival thanks to this combo treatment approach every year [40]. Immunotherapy, particularly ICIs, has transformed the way solid tumor malignancies are treated. The strongest ICI results for breast cancer are now available for TNBC [13].

Immuno-monotherapy

Patients with metastatic triple-negative breast cancer (mTNBC) were the first to undergo immunotherapy safety and efficacy assessments. Pembrolizumab monotherapy was reported to have greater response rates when administered as the first line of treatment for PD-L1-positive (PD-L11) illness [10]. When used alone for mTNBC, atezolizumab and avelumab showed some promise in treating PD-L1-positive cases as a first-line treatment [11]. According to the findings of these investigations, patients with PD L11 breast cancer may benefit more from ICI monotherapy, but with a limited survival advantage [13].

Immunotherapy and Chemotherapy

The available choices for chemotherapy include carboplatin plus gemcitabine, paclitaxel, or nab-paclitaxel. Research findings indicated that the pembrolizumab group experienced a higher rate of immune-related adverse events (irAEs) at 26.5% than the placebo group did, with 5.3% of the pembrolizumab group experiencing grade ≥ 3 irAEs and 0% of the placebo group experiencing them [10,13]. The three most frequent irAEs in the atezolizumab group were rash, hypothyroidism, and hepatitis, with a rate of 57.3% in the atezolizumab group and 41.8% in the placebo group [13]. Hepatitis was the most frequent grade ≥ 3 irAE, occurring in 7.5% of participants in the atezolizumab group compared to 4.3% in the placebo group [13]. As a result of these findings, atezolizumab in combination with nab-paclitaxel was first approved by regulators more quickly in the US and received complete approval globally for mTNBC [13].

ADCs

Preclinical evidence suggests that combining ADCs with ICIs could enhance their effectiveness. This potential boost in efficacy is attributed to increased production of neoantigens, higher PD-L1 expression, and direct activation of dendritic cells [12]. Trials including trastuzumab-deruxtecan and durvalumab, ladir-atuzumab vedotin and pembrolizumab, sacituzumab govitecan with pembrolizumab and atezolizumab in mTNBC, and a second TROP2 an ADC, datopotamab-deruxtecan and durvalumab, are presently underway [10,11,12,13].

Dual Immunotherapy

Examining combined CTLA4 and PD-L1 inhibition in the management of TNBC has drawn more attention. In a trial with 17 women who had metaplastic breast cancer and had already tried several treatments, the combination therapy with nivolumab (a PD-1 inhibitor) and ipilimumab (a CTLA-4 inhibitor) resulted in an overall response rate of 18% [13]. Of these patients, eight experienced irAEs, with three of them being adrenal insufficiency. More research is being conducted to assess the combination of ipilimumab and nivolumab, as well as durvalumab and tremelimumab (an additional CTLA-4 inhibitor), in HER2-positive breast cancer patients [41].

Immunotherapeutic Strategies Under Development

Treatment-effective poly (ADP-ribose) polymerase (PARP) inhibitors are available for individuals with germline mutations in BRCA1 or BRCA2 (gBRCA). Based on preclinical studies, PARP inhibitors have the potential to enhance the host's immune response to tumors by inducing neoantigens through DNA damage, inducing interferon production via the STING pathway, and elevating the expression of PD-L1 [13]. Novel treatments for breast cancer are being researched, vaccinations against the disease, and adoptive cell therapies like CAR-T and T cell receptor therapy [14]. Including gene therapy, promising outcomes were also observed in the inhibition of breast cancer cell growth and development when microRNA was used in anti-cancer therapy. As far as we are aware, MRX34 is among the first miRNA substitution medications (miR-34a), and it is currently undergoing clinical studies.

Determining an appropriate response evaluation is a challenge in the development of immunotherapy since the pattern of responses to ICIs differs from that of chemotherapeutic drugs [42]. The Immune Response Evaluation Criteria in Solid Tumors (iRECIST) were developed to better assess the benefits of immunotherapy, but most clinical trials still use the traditional RECIST criteria [43]. The recent GeparNUEVO study highlights this issue [44]. Consequently, a pCR after NACT in breast cancer is often used as a substitute endpoint for long-term outcomes. However, this may not effectively capture long-term immune memory responses, which are crucial for maintaining therapeutic effects and preventing relapses [42].

Possible complications and adverse effects

Immunotherapy has recently emerged as a transformative approach to breast cancer, but it is not without complications and adverse effects. irAEs that are a consequence of treatment with ICIs are referred to as acute irAEs, those that occur after completion of treatment are called delayed irAEs, and those that are persistent after 12 weeks of ICI discontinuation are chronic irAEs [45]. The most frequently observed irAEs are dermatologic, gastrointestinal, hepatic, endocrine, and pituitary and are most meticulously documented in the treatment cases of patients administered ICIs due to their clinical popularity [46,47]. The incidence and pattern of late irAEs appear similar to early irAEs and 85% irAEs occur within the first six months of treatment [48]. Long-term use of immunotherapy was also found to be associated with more frequent and chronic irAEs like endocrinopathies, immunologic, neurological, cardiovascular, and ophthalmological effects [45-48]. Studies found that irAEs were linked to delays in receiving adjuvant radiation therapy, but they did not correlate with postoperative complications or delays in surgery [49]. While biomarkers for predicting irAEs are not yet available in clinical practice, retrospective observational studies have identified certain clinical characteristics that are associated with an increased risk of irAEs such as female gender, genetic factors, specific microbiome configurations, and circulating biomarkers that are associated with an increased risk of irAEs.

There are multiple endocrine complications linked to the use of immunotherapy including a higher risk of hypothyroidism, hyperthyroidism, hypophysitis, and adrenal insufficiency compared with the control group [46-51]. This frequently presents as nonspecific symptoms like nausea, headache, fatigue, and vision changes [47]. Hematologic irAEs, such as asymptomatic cytopenia, immune thrombocytopenic purpura, autoimmune hemolytic anemia, acquired hemophilia, and disseminated intravascular coagulopathy, can occasionally occur during treatment with ICI combination therapy. The severity of these effects can range from mild to severe [52]. Pneumonitis is an unusual but fatal complication that is asymptomatic. It is usually identified on routine computed tomography imaging. The first-line treatment is corticosteroids while stronger immunosuppressors (such as infliximab and cyclophosphamide) can be used in refractory cases [47].

Cardiovascular side effects include arterial and pulmonary hypertension, supraventricular and ventricular arrhythmias, systolic and diastolic cardiac dysfunction, and coronary artery diseases due to different molecular pathways that are still not well understood [53]. Cutaneous toxicity can be classified into several categories: inflammatory reactions, neoplastic conditions, severe reactions (like Stevens-Johnson syndrome or toxic epidermal necrolysis), connective tissue diseases, antibody-mediated effects, hair-related issues, and other occasional reactions. The most common presentation of the dermatologic side effects of ICIs in combination therapy for breast cancer is rash and pruritus, experienced by around 40% of the patients [47,52]. The time to onset of specific cutaneous irAE may provide a clue to the correct diagnosis [47]. Diarrhea and colitis are the most frequent gastrointestinal irAEs, typically starting around the sixth or seventh week of treatment, with recovery generally occurring by the 10th week [46,47,52,54]. Hepatotoxicity is relatively rare presenting as elevated liver enzymes between eight and 12 weeks after treatment initiation [47].

**Figure 2:**
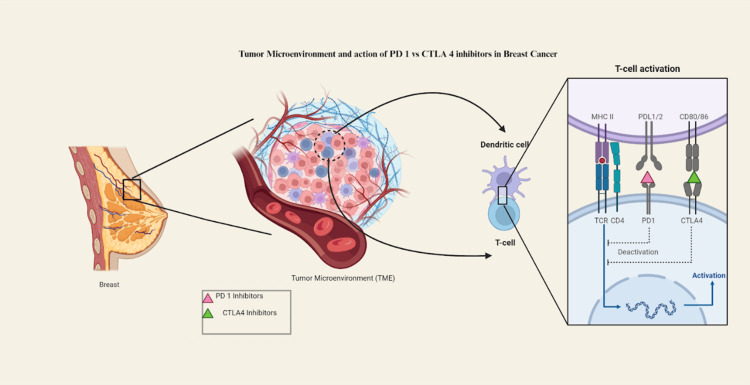
Tumor microenvironment and action of PD 1 versus CTLA 4 inhibitors in breast cancer Figure made by authors using BioRender.com/p37w814

The newer novel therapies like PD-1 inhibitors like pembrolizumab are found to cause fewer irAEs compared to the older anti-CTLA 4 inhibitors (Table 3) [46]. Older therapies like trastuzumab have been associated with congestive heart failure, susceptibility to respiratory infections, gastrointestinal effects like diarrhea (and others), skin rashes, and constitutional symptoms like fatigue [50,54]. Studies on the safety and effectiveness of using the programmed cell death protein 1 (PD-1) inhibitor for patients with advanced TNBC have shown that, while adverse effects can occur, they are generally limited to constitutional symptoms such as joint pain, fatigue, nausea, and muscle pain (Figure 2) [55,56].

**Table 3:** Comparison of adverse effects in older versus newer immunotherapies

Immune-related adverse effect	Older immunotherapies (e.g., MAbs like trastuzumab and CTLA 4-like ipilimumab)	Newer immunotherapies (e.g., PD L1 inhibitors like pembrolizumab)
Constitutional symptoms	Common	Less common
Gastrointestinal toxicity (diarrhea)	Common	Less frequent
Dermatologic toxicity	Common (e.g., rash, pruritus)	Less frequent
Cardiovascular toxicity (heart failure)	Higher incidence	Lower incidence

The stromal network in the breast tumor microenvironment, which is characterized by a dense collagen extracellular matrix, not only restricts penetration of systemically administered drugs but also promotes tumor progression and metastasis. This can ultimately result in drug resistance and treatment failure [46]. Previous literature has extensively focused on the negative effects of immunosuppressive agents. However, there has been limited research on managing the adverse immune effects that might arise when immunotherapy is combined with other treatments. Furthermore, there is a lack of consensus on the approach to take for patients who require second-line immunosuppressive therapy [46,52].

The primary treatment against irAEs is systemic corticosteroid administration [45], although the associated immunosuppression may compromise the antitumor response [38]. Additional treatment options include supportive care, additional immunosuppression, and considering treatment delay or discontinuation (Table 4) [45]. T-cells are thought to play a key role in most irAEs. Hence, treatments like mycophenolate, an inosine monophosphate dehydrogenase (IMPDH) inhibitor that depletes guanine nucleotides in T cells and B cells, can be used. This helps to suppress the immune response and antibody production. Other treatments, such as calcineurin inhibitors and methotrexate, can also be used to control T-cell activity, especially in cases where steroids are not effective [47]. It is important to highlight the fact that for irAEs such as neurologic, hematologic, and cardiac toxicity, the only definitive treatment is to terminate immunotherapy [52]. Refractory skin irAEs can be managed with secukinumab, an anti-IL17 antibody, steroid-refractory colitis with infliximab, a monoclonal antibody against the tumor necrosis factor (TNF), some other therapies like tocilizumab and rapamycin for GI or hematological irAEs and allograft tolerance for solid organ transplantation respectively are still experimental and are associated with various other adverse effects [47].

**Table 4:** Some management strategies for immune-related adverse events (irAEs)

Immune-related adverse effect type	First-line treatment	Second-line treatment	Notes
Gastrointestinal (e.g., colitis)	Corticosteroids	Infliximab	May warrant close monitoring
Dermatologic (rash, pruritus)	Topical steroids	Systemic corticosteroids	Secukinumab used for refractory cases
Hepatotoxicity	Corticosteroids	None	May require liver function (LFT) monitoring
Cardiovascular	Discontinuation of immunotherapy	None	Requires regular monitoring and evaluation.
Endocrinopathies	Hormone replacement therapy	None	Mainly includes management of hypo or hyperthyroidism, hypophysitis, and others

Considering manipulating the immune system to fight tumors can as with immunotherapy result in side effects distinct from those seen with traditional chemotherapy, these irAEs need special care. Despite these adverse effects being generally less severe with the use of newer immunotherapy regimens, managing them often requires advice from a team of specialists [45]. Thus, looking for an effective strategy for irAEs' management is necessary and clinicians must raise awareness of the management of adverse immune reactions [52].

Way forward

Despite the progress made over the years in the management of breast cancer with the advent of immunotherapy and molecular markers, there are still challenges and aspects to be elucidated for the future. Some of the questions that persist to this moment are: which immunotherapy regimen is the ideal based on the molecular characterization of the cancer? Is immunotherapy relevant to HER+ cancers? Which adjuvant regimen should be used when there is a partial response to neoadjuvant treatment? [20,42,57-59] Questions that may be answered with the upcoming and promising therapies currently in research, such as the use of bispecific antibodies (bsAbs), CAR-based immunotherapy, and dual checkpoint inhibition [60-63]. Likewise, research remains to establish the most effective strategies for managing irAEs [42].

There is also a lack of clarity in some aspects related to surgery. Currently, there is no consensus on what type of breast surgery or if it should be performed if there is a pCR after neoadjuvant therapy. Moreover, some authors question whether it is necessary to perform axillary surgery, especially if the patient has limited involvement in the SLN and is planning to receive adjuvant therapy [20,57].

However, what seems to be a challenging priority, especially when talking of a cold tumor, is the deeper characterization of the breast cancer microenvironment, immune landscape, and immunogram, which may help to discover new disease biomarkers and treatment targets [7]. That will allow an individualized and specific treatment for each type of neoplasm and, consequently, improve the prognosis and future of the patients.

## Conclusions

In recent years, we have seen the advancement in new strategies for breast cancer treatment, with immunotherapy being one of the most promising, especially when it is combined with surgery. Investigation is warranted to try to characterize the breast cancer microenvironment. This will allow treatment strategies to focus more on individualization and de-escalation of therapy based on tumor biology, novel biomarkers, and tumor spread. There are still promising new strategies surrounding immunotherapy in study and development, which promise to change not just patients' prognosis in each state of the disease and sub-type, but also the way that surgery is performed.
